# Supplementary material for: Ticagrelor vs Clopidogrel in addition to Aspirin in minor ischemic stroke/ transient ischemic attack—Protocol for a systematic review and network meta-analysis
Source: PLoS One. 2021 Apr 28;16(4):e0250553. doi: 10.1371/journal.pone.0250553 (PMC8081237; doi:10.1371/journal.pone.0250553)
Supplement: S2 File — (DOCX) [file pone.0250553.s003.docx]

**Supporting information – Supplemental Materials 1: Search Strategy**

1. exp stroke/

2. brain ischemia/ or ischemic attack, transient.mp. [mp=title, abstract, original title, name of substance word, subject heading word, floating sub-heading word, keyword heading word, organism supplementary concept word, protocol supplementary concept word, rare disease supplementary concept word, unique identifier, synonyms]

3. (brain isch?em* or cerebral infarct*).tw.

4. ((brain or cerebral) adj isch?em*).tw.

5. stroke*.tw,kw.

6. ((brain or cerebral) adj infarct*).tw.

7. (brain infarct* or cerebral infarct*).kw.

8. transient isch?em* attack*.tw,kw.

9. 1 or 2 or 3 or 4 or 5 or 6 or 7 or 8

10. Ticagrelor/

11. (Ticagrelor or azd6140 or azd 6140 or brilinta).tw,kw.

12. 10 or 11

13. 9 and 12

14. Clopidogrel/

15. (Clopidogrel or pcr4099 or pcr 4099 or plavix).tw,kf.

16. aspirin/

17. (aspirin or acetylsalicylic acid).tw,kw.

18. (random* or placebo).mp. or trial.ti.

19. 14 or 15

20. 9 and 19

21. 13 or 20

22. 16 or 17

23. 18 and 21 and 22

24. limit 23 to "therapy (best balance of sensitivity and specificity)"

*Note that there is no limit like line 24 in Cochrane, so only the first 23 were used.
